# Effect of fire on the palatability of plants in an African woodland savanna: varying impacts depending on plant functional groups

**DOI:** 10.7717/peerj.12721

**Published:** 2022-01-19

**Authors:** Caroline Stolter, David F. Joubert, Nekulilo Uunona, Elise Nghalipo, Vistorina Amputu, Annika M. Felton

**Affiliations:** 1 Department of Animal Ecology and Conservation, Hamburg University, Hamburg, Germany; 2 Faculty of Natural Resources and Spatial Sciences, Namibia University of Science and Technology, Windhoek, Namibia; 3 Institute of Evolution and Ecology, University of Tuebingen, Tuebingen, Germany; 4 Southern Swedish Forest Research Centre, Swedish University of Agricultural Sciences, Alnarp, Sweden

**Keywords:** Grassland, Rangeland, Feed, Plant response, Fire, Plant quality, Herbivores, Grasses, Trees, Food quality

## Abstract

Fire and herbivores are two important drivers of changes in vegetation composition, quality and dynamics and both are highly related to each other. Herbivores are known to respond to fire both in terms of foraging decisions and distribution. However, little is known about the actual changes in plant chemistry following a fire event and how long these changes will last. We investigated the effect of fire on two different plant functional groups (grasses and woody species) in a woodland savanna of southern Africa. We studied chemical compounds known to be important for palatability of five perennial grass and seven woody species (trees and shrubs) common in the woodland savanna and known to be utilized by herbivores. We wanted to know if plant chemistry differs between a recently burned site (burned 2 years ago) and a control site, burned 16 years ago, and if grasses and woody species show similar relative differences between sites (*i.e.*, the plants’ response to fire). We found a clear difference in chemical composition patterns between the plant functional groups, with an almost homogenous response to fire among woody species, but higher variability in response among grass species. Furthermore, we found that woody species maintained a higher nutritional value even 2 years after burning, whereas grasses did not show clear differences among the two investigated sites. Hence, few years after burning, woody plants might still serve as an attraction for herbivores, especially browsers, in contrast to grasses. The knowledge about these differences between the two functional groups in response to fire is beneficial for the development of management strategies for large herbivores whether domestic or wild.

## Introduction

Savannas cover large areas of the sub-tropical and tropical part of the world. In these regions, one fifth of the human population as well as the majority of the world’s domestic and wild large herbivores are found (Graz, 2008). Savannas are characterized by dominating grassland interspersed with a discontinuous cover of trees and shrubs. Next to climate and soil characteristics, fire and herbivory are important factors for the maintenance of these ecosystems (Skarpe, 1992; Van Langevelde et al., 2003; Bond & Keeley, 2005; Anderson, Fuhlendorf & Engle, 2006). As components of the natural disturbance regime of savannas, both fire and herbivory affect primary production, ecological processes and functions, resulting in changes to plant species distribution and composition (Scholes & Archer, 1997; Olff & Ritchie, 1998; Higgins, Bond & Trollope, 2000; Roques, O’Connor & Watkinson, 2001; Anderson et al., 2007; Sankaran, Ratnam & Hanan, 2008; Keeley et al., 2011; Benthien et al., 2018). Any anthropogenic changes of naturally occurring fire regimes or the utilization of man-made fires as a management tool, are likely to influence savanna vegetation dynamics and cause changes in ecosystem function (Bird & Cali, 1998; Guyette, Muzika & Dey, 2002; Joubert, Rothauge & Smit, 2008; Joubert, Smit & Hoffman, 2012; Lohmann et al., 2014; Trollope et al., 2014).

While fire locally affects patterns of vegetation composition, vegetation composition in turn influences the distribution of herbivores (Senft et al., 1987; Fryxell & Sinclair, 1988; Uunona, 2014). In a given habitat the distribution of herbivores, whether leaf chewing insects or free ranging large herbivores, is to some extent driven by the chemical composition of specific food plants (*e.g.*, Ben-Shahar & Coe, 1992). Vegetation offers a multidimensional feeding environment of different nutrients and anti-nutritional compounds to herbivores. The animal has to choose which diet satisfies its nutritional needs (*e.g.*, amount of protein and fiber), while avoiding possible toxic or anti-nutritional effects of plant secondary metabolites (PSMs) (Villalba, Provenza & Bryant, 2002). Their food selection is challenging as the concentration of chemical compounds varies among different plant species (inter-specific variation; Rogosic et al., 2006), between different specimens of the same species (intra-specific, Stolter et al., 2005), and even within a plant individual (intra-individual differences; Nordengren, Hofgaard & Ball, 2003; Stolter, 2008).

The chemical composition of plants varies also between plant functional groups, such as grasses and woody plant species. Generally, grasses are known to be high in fiber, but compared to leaves of woody species, relatively low in nitrogen and secondary plant metabolites (Van Soest, 1994). Herbivorous insects have co-evolved with plants and have reached a high level of adaptation to consume plant material, *e.g.*, the use of specific plant secondary metabolites for own defense mechanisms (Zunjarrao et al., 2020). Mammalian herbivores are also highly adapted to the differences in the chemical composition of their food and are usually divided in three different feeding guilds (grazer, browser and intermediate type). In contrast to browsers, grazers are highly adapted to a fiber rich diet, and can therefore subsist on a grass-dominated diet (Gordon & Illius, 1994). The fermentation of hemicellulose and cellulose provides up to 80% of the required energy for these animals (Barboza, Parker & Hume, 2008). On the other hand, browsers can tolerate higher amounts of plant secondary metabolites (Iason & Villalba, 2006). For all feeding guilds lignin is least digestible (Van Soest, 1994).

Changes in concentration of different plant compounds are related to temperature, water availability, light and soil characteristics, but also herbivory (Bathurst & Mitchell, 1958; Bryant, Chapin & Klein, 1983; Wilson, 1983; Close & McArthur, 2002; Scogings, Hjältén & Skarpe, 2011). Therefore, the concentrations of different compounds will change with seasons but also with different habitat types and/or site conditions. For example, plants of one species on fertile soil can be higher in quality (*e.g.*, higher in nitrogen concentration, lower in fiber and PSM concentration) than conspecifics on nutrient poor soils (Bryant, Chapin & Klein, 1983; Blair, 1997; Ball, Danell & Sunesson, 2000; Kraus, Zasoski & Dahlgren, 2004; Stolter, Ball & Julkunen-Tiitto, 2013). Fire can cause dramatic changes in these factors (*e.g.*, in soil characteristics) with pronounced consequences for the chemical composition of plants and the feeding decisions of the associated herbivore community. The effects of fire on soils are complex, as biomass, down to the topsoil layer, can be removed causing severe changes in plant and microbial populations (Knicker, 2007). Well known are the fertilizing effects of fire due to mineral rich ash remaining after combustion which in turn increases pH (Ballard, 2000; Grogan, Burns & Chapin, 2000; Certini, 2005). Therefore, an increased post-fire growth is frequently reported, but depends on the plant species in focus (Van de Vijver, Poot & Prins, 1999; Gignoux, Clobert & Menaut, 1997; Giardina & Rhoades, 2001; Rieske, Housman & Arthur, 2002; Schutz, Bond & Cramer, 2009). A short-term increase in nutrient mineralization and availability (Hobbs & Schimel, 1984; Ojima et al., 1994) may also lead to an increase in nitrogen concentration in the post-fire regrowth (Van de Vijver, Poot & Prins, 1999). At the same time, plants have to compensate for tissue loss, and therefore enhance their protein metabolism (*e.g.*, to gain higher photosynthetic rates; Evans & Clarke, 2019). This may lead to an enhanced growth rate of new tissue and therefore a lower concentration of PSMs (Growth Differentiation Balance Hypothesis, Herms & Mattson, 1992; Hattas, Scogings & Julkunen-Tiitto, 2017; Scogings, 2018), as the new growth of leaves and the allocation of carbon based PSMs compete for available products of the photosynthesis (Carbon Nutrient Balance Hypothesis, Bryant, Chapin & Klein, 1983; Koricheva et al., 1998). Furthermore, the replacement of old leaves with new regrowth might lead to an overall enhancement of food quality in a recently burned area as new leaves will be easier to digest because of less structural carbohydrates (*e.g.*, less lignin, Mediavilla et al., 2014).

We studied the effects of fire on plant palatability of five different grass species and seven woody species common in the woodland savanna in southern Africa. The plant species in question are known to be selected as forage by large wild herbivores (Uunona, 2014) but little is known about the post-fire changes in palatability in these plant species, especially concerning woody plants. We analyzed the plant material of woody species and grasses for nitrogen, different fiber fractions and ash (as a proxy for mineral content) of two neighboring sites (one burned 2 years ago, and one control site, burned 16 years ago). In addition, we analyzed plant secondary metabolites (condensed tannins and total phenolics) in samples from woody plants. We chose to analyze these plant compounds as they are relevant for the food choice of different wild ruminants (Iason & Villalba, 2006; Barboza, Parker & Hume, 2008; Felton et al., 2018; Stolter, 2018).

Because grasses and woody species were subjected to similar abiotic factors at our study sites, our hypothesis was that species belonging to both plant functional groups show similar relative differences in plant chemical composition between the recently burned and the control site (which will hereafter be referred to as the plants’ response to fire). Specifically, we expected nitrogen and ash to be higher, and fiber compounds to be lower, in grasses and leaves from woody species available in the recently burned area compared to the control site. Furthermore, we expected PSMs to be lower in plant material of woody species of the recently burned site. In combination, this would lead to a generally higher nutritional quality and palatability for herbivores shortly after fire than after 16 years. Short term effects of fire on ground vegetation, especially grasses, have been reported by several studies (*e.g*., Ojima et al., 1994; Moe & Wegge, 1997; Van de Vijver, Poot & Prins, 1999; Anderson et al., 2007; Parrini & Owen-Smith, 2010; Mbatha & Ward, 2010). Here we provide a rare complement, by investigating the plant material with a longer time since last fire, and comparing different plant functional groups important for savanna herbivores.

## Materials and Methods

The project was carried out under the frame of SASSCAL (Southern African Science Service Center for Climate Chance and Adaptive Land Management). The Ministry of Environment, Forestry and Tourism of Namibia, Windhoek, allowed the fieldwork to be conducted in Waterberg Plateau Park (WPP). The National Botanical Institute of Namibia gave permission for the transfer of the plant material. Sampling took place at the Waterberg Plateau Park (WPP), a Table Mountain, which is located in central Namibia (20°37′S, 17°08′E & 20°11′S, 17°26′E) with an elevation of 1,550–1,850 m a.s.l.. Information about the fire history, the sites, the vegetation and soil characteristics is well elaborated in Joubert et al. (2018), Amputu, Joubert & Mapaure (2019) and Nghalipo et al. (2018) respectively. The mean precipitation during the rainy season October 2014–April 2015, was 300 mm with the highest precipitation in November and December (724 mm and 714 mm; http://www.sasscalweathernet.org). The WPP has woodland savanna and Kalahari woodland vegetation on sandy soils (Kalahari sands). The soil is nutrient poor with a low content of clay (for details see Nghalipo et al., 2018 and Table 1). Dominant woody species are *Terminalia sericea, Acacia ataxacantha (Senegalia ataxacantha), Acacia fleckii (Senegalia fleckii), Combretum collinum, Combretum psidioides, Grewia avellana, Grewia flavescence, Grewia retuinervis, Grewia flava, Burkea africana, Croton gratissimus, Peltophorum africanum, Ochna pulchra, Philenoptera nelsii, Dombeya rotundifolia, Ximenia americana* and *Ximenia caffra* (Amputu, Joubert & Mapaure, 2019). While, dominant perennial grasses are *Eragrostis pallens, Brachiaria nigropedata, Digitaria seriata, Panicum kalahariensis, Aristida stipitata*, *Stipagrostis uniplumis* and *Eragrostis jeffreysii* (Amputu, Joubert & Mapaure, 2019).

**Table 1:** Soil characteristics excerpted from Nghalipo et al. (2018).

	Control site	Recently burned site
SOC (%)	0.39	0.39
Total *N* (%)	0.13	0.07
P (ppm)	1.67	1.44
K (ppm)	38.9	42.6
Na (ppm)	9.30	30.5
Mg (ppm)	20.4	27.6
Ca (ppm)	42.0	60.0

**Notes:**

Mean values of soil organic carbon (SOC) and nutrient concentrations in the top soil (0–10 cm depth) of the recently burned site (*n* = 6) and the control site (*n* = 6).

Sampling occurred 1 year before this study (*i.e.*, 1 year after burning).

The WPP is divided into different fire zones with known fire history (Joubert et al., 2018). This knowledge offers the opportunity for a comparison of different plant species between sites with different time since last fire. In addition to the known fire history, the study sites are similar as they have similar climate and soil conditions (Nghalipo et al., 2018) and support the same vegetation (Amputu, Joubert & Mapaure, 2019), allowing the effect of fire to be investigated.

### Plant sampling

We sampled plants in a recently burned site (a lightning ignition fire burned 2 years before sampling, thereby the whole site was burned) and in a site which had not burned for 16 years before sampling. Both sampling sites are about the same size (30 km²) and in immediate vicinity to each other. The sites are only divided by a sand road, which serves as a firebreak. Sampling took place in April 2015, at the end of the rainy season. We chose this time of the year as the yearly plant growth starts with the rainy season and we aimed to collect newly grown leaves from both functional groups. We collected five different perennial grass species: *Aristida stipitata* (AS), *Brachiaria nigropedata* (BN), *Digitaria seriata* (DS), *Eragrostis jeffreysii* (EJ), *Stipagrostis uniplumus* (SU); and seven different woody species: *Acacia ataxacantha* (AA) = *Senegalia ataxacantha*, *Bauhinia petersiana* (BP), *Burkea africana* (BA), *Combretum psidioides* (CP), *Grewia flava* (GF), *Philenoptera nelsii* (PN), *Terminalia sericea* (TS). Thereof, all grass species are C4 type (Osborne et al., 2014; Simpson et al., 2020), and *B. petersiana*, *B. africana, and A. ataxacantha* are nitrogen-fixating tree/shrub species. All plant species are known to be utilized by wild large herbivores in this area, *e.g., Giraffa camelopardalis* (giraffe), *Syncerus caffer* (African buffalo), *Taurotragus oryx* (eland), *Raphicerus campestris* (steenbok), *Tragelaphus strepsiceros* (greater kudu), *Hippotragus equinus* (roan antelope) and *Hippotragus niger* (sable antelope) (Uunona, 2014). For each plant species, we collected six replicates (*n* = 6 per species) randomly in the recently burned site and the control site. We sampled plants with a minimum of 20 m distance between each other. For comparability we sampled only pristine green shoots from grasses in the middle of the tussock, and for woody plants we sampled only undamaged leaves of new shoots at the top 20 cm of the shoots. For this, we used only the first distal leaves from 7–15 northern side shoots (min. height: 80 cm, max. height: 170 cm).

### Chemical analyzes

Samples were air dried and ground to pass a 1 mm sieve. All plant samples were analyzed for nitrogen using the Kjeldahl procedure (Association of Official Analytical Chemists, 1984). The content of different fiber fractions, including lignin and ash content, was determined with an ANKOM Fiber Analyzer (ANKOM Technology, Macedon, New York; Ortmann et al., 2006). We calculated the mathematical difference of sample dry weight minus neutral detergent fiber (NDF) to gain the concentration of soluble cellular content (SCC). In addition, to gain the concentration of hemicellulose we calculated the concentration of NDF minus the concentration of acid detergent fiber (ADF), and ADF minus acid detergent lignin (ADL) to gain the concentration of cellulose. ADL is hereafter referred to as lignin. Ash was determined after combustion of the ADL samples (for details see Ortmann et al., 2006). For samples of woody species we additionally analyzed the concentrations of condensed tannins (CT) with the Butanol-HCl method, Oates, Swain & Zantovska, 1977, extracted in 50% methanol and total phenolics (TP) by using the Folin-Ciocalteaus method (extracted in boiling water, Folin & Ciocalteau, 1927). We did not conduct these analyzes (CT, TP) for grass species, as grasses are low in concentrations of PSMs and different in the composition of specific phenolic compounds compared to woody plant species (Iason & Villalba, 2006), therefore a comparison between both plant functional groups in CT and TP analyzed by our methods would be misleading.

### Statistics

Statistical analyzes were performed with SPSS (PASW Statistics version 18, PASW 2010). To compare the difference in concentrations of plant chemical compounds between woody species and grasses, we conducted independent t-test or Mann-Whitney U-Test. To compare the overall differences (all species) in concentrations between the burned and the control site, we conducted either Wilcoxon or paired sampled t-test. For all these calculations the mean value of each species was used. For the comparison of concentrations between CT and TP we used the Wilcoxon Test.

We used a general linear model to test for the effect of burning, plant functional group and species on the chemical composition. We used the chemical compound as dependent variable, the interaction between plant functional group and burned status as a fixed factor and species as random factor. All variables were tested for deviation of homogeneity of variance (Levene’s test). We did not include CT and TP in our modelling approach, as these compounds were only analyzed for woody species.

To gain a deeper knowledge on the chemical pattern we tested for intra-specific differences. Depending on the distribution of the data, we used independent t-test or Mann-Whitney U-Test to analyze differences in the chemical composition of each species between sites (burned *vs* control). Furthermore, in order to visualize the differences in plant response, we used principal component analyzes to group plant species according to their differences in means between recently burned and control site into principal components (PCA; Stolter et al., 2010). For this we calculated, for each plant species, the differences between the mean concentration of a given nutritional variable in material sourced from the recently burned *vs* the control area and grouped the results using PCA. Firstly, we plotted all plant species and their relative differences in means with regards to the primary compounds. Secondly, we plotted these relative differences in means for the two plant functional groups (grasses or woody species) separately, including PSMs which were only measured for woody species.

## Results

For details of all concentrations and compounds see Tables 2–4. Generally, the mean concentration of nitrogen and soluble cell content (SCC) in leaves of woody species was significantly higher compared to grasses (N: t = 8.14, *P* ≤ 0.001; SCC: z = 10.21, *P* ≤ 0.001 for both *n* = 12 (means of five grass and seven woody species)). For hemicellulose and cellulose this pattern was inverse (hemicellulose: t = 32.09, *P* ≤ 0.001; cellulose: t = 25.08, *P* ≤ 0.001; *n* = 12). There were only slightly, but significantly higher concentrations of lignin in the leaves of woody species compared to grasses (t = 12.04, *P* ≤ 0.001; *n* = 12). Generally, the concentration of ash was similar for grasses and woody species (t = 0.62, *P* = 0.533, *n* = 12). Among woody species, we found higher concentrations of total phenolics compared to condensed tannins (W = 3,570, *P* ≤ 0.001, *n* = 14).

**Table 2:** Concentrations (%) of nitrogen and ash in leaves of woody species and grasses found at two different sites.

	PFG	Control		Burned				
		Mean	Std. dev.	Mean	Std. dev.	Mean Diff.	T/Z/W	*P*
**Nitrogen**								
All	–	1.89	0.97	2.06	1.14	–	*52	0.307
Grass	G	1.27	0.41	1.30	0.45	–	−0.122	0.909
Woody species	T	2.35	1.02	2.61	1.19	–	−1.287	0.246
BN	G	1.93	0.37	2.07	0.42	0.14	−0.619	0.550
AS	G	1.01	0.07	1.12	0.12	0.12	−1.967	0.077
DS	G	1.27	0.3	1.15	0.26	−0.11	−0.320z	0.749
EJ	G	1.26	0.08	1.24	0.11	−0.02	0.308	0.765
SU	G	0.86	0.09	0.91	0.15	0.06	−0.769	0.460
TS	T	1.38	0.12	1.64	0.2	0.27	−2.820	**0.018**
AA	T	2.12	0.11	2.84	0.56	0.72	−3.077	**0.025**
PN	T	4	0.39	5.05	0.36	1.05	−4.798	**0.001**
GF	T	2.83	0.35	2.61	0.23	−0.23	1.326	0.214
CP	T	1.51	0.108	1.8	0.106	0.28	−4.544	**0.001**
BA	T	1.4	0.1	1.67	0.15	0.27	−3.581	**0.005**
BP	T	3.18	0.38	2.64	0.14	−0.54	3.262	**0.016**
**Ash**								
All	–	3.73	1.55	3.84	1.40	–	−0.29	0.778
Grass	G	3.77	1.80	3.62	1.41	–	0.158	0.882
Woody species	T	3.71	1.50	4.01	1.49	–	−6.730	**0.008**
BN	G	6.02	2.11	5.1	0.61	−0.92	−0.641z	0.522
AS	G	2.7	0.24	2.43	0.23	−0.27	2.008	0.072
DS	G	5.35	1.18	5.03	0.64	−0.32	0.585	0.572
EJ	G	2.91	0.36	3.43	1.43	0.4	−0.32	0.749
SU	G	1.88	0.12	2.01	0.64	0.22	−0.826	0.444
TS	T	2.71	0.29	3.15	0.46	0.44	−1.976	0.076
AA	T	3.75	0.32	4.12	0.44	0.38	−1.702	0.120
PN	T	4.18	0.67	4.36	0.25	0.18	−0.608	0.557
GF	T	6.58	0.53	6.93	0.77	0.35	−0.908	0.385
CP	T	3.16	0.25	3.48	0.46	0.32	−1.492	0.166
BA	T	1.78	0.24	2.13	0.57	0.35	−1.283z	0.199
BP	T	3.8	0.47	3.91	0.49	0.1	−0.369	0.720

**Notes:**

Mean diff, Differences in means between burned and control sites; PFG, Plant functional group with G, Grasses; T, Trees/woody species; grass species: AS, *Aristida stipitata*; BN, *Brachiaria nigropedata*; DS, *Digitaria seriata*; EJ, *Eragrostis jeffreysii*; SU, *Stipagrostis uniplumus*; Tree species: AA, *Acacia ataxacantha*; BA, *Burkea africana*; BP, *Bauhinia petersiana*; CP, *Combretum psidiodes*; GF, *Grewia flava*; PN, *Philenoptera nelsii*; TS, *Terminalia sericea*.

All, All species; T, T-test; Z, Mann-Whitney-U-Test (indicated by “z”), Wilcoxon Test by*.

Significant differences (*P*) are indicated in bold.

**Table 3:** Concentrations (%) of different fiber compounds in leaves of woody species and grasses found at two different sites.

	PFG	Control		Burned				
		Mean	Std. dev.	Mean	Std. dev.	Mean Diff.	T/Z/W	*P*
**SCC**								
All	−	45.94	19.64	47.29	20.21	**-**	*51	0.347
Grass	G	25.02	6.11	25.24	5.72	**-**	−0.059	0.956
Woody species	T	60.88	7.55	63.04	5.69	**-**	−1.429	0.203
BN	G	32.26	2.51	32.1	2.02	−0.16	0.122	0.906
AS	G	20.53	1	21	0.98	0.46	−0.809	0.437
DS	G	31.04	1.92	30.77	2.8	−0.26	0.189	0.854
EJ	G	21.8	0.63	22.16	0.69	0.36	−0.932	0.373
SU	G	19.49	0.96	20.16	1.93	0.67	−0.760	0.465
TS	T	67.85	1.6	66.69	2.63	−1.16	0.923	0.378
AA	T	69.52	4.86	72.43	4.37	2.91	−1.090	0.330
PN	T	48.80	4.52	58.81	2.93	10.01	2.560z	**0.010**
GF	T	54.41	2.67	56.71	3.77	2.29	−1.215	0.252
CP	T	58.94	2.38	57.63	2.88	−1.31	0.860	0.410
BA	T	60.59	1.47	63.88	2.93	3.29	−2.460	**0.040**
BP	T	66.07	3.88	65.17	2.13	−0.91	0.500	0.628
**Hemicellulose**								
All	–	22.62	11.16	22.39	11.68	–	*46	0.583
Grass	G	34.56	3.35	34.93	4.14	–	−0.936	0.402
Woody species	T	14.10	4.14	13.43	3.73	–	1.249	0.258
BN	G	36.10	1.60	37.52	1.47	1.43	−1.604	0.140
AS	G	36.37	0.32	36.57	0.77	0.21	−0.642z	0.521
DS	G	30.41	2.36	30.32	1.41	−0.09	0.08	0.938
EJ	G	38.26	0.75	39.48	0.71	1.21	−2.873	0.017
SU	G	31.68	0.51	30.78	1.71	−0.9	1.233	0.265
TS	T	10.46	1.28	9.98	1.84	−0.48	−0.962z	0.336
AA	T	14.1	2.87	12.66	2.7	−1.45	0.898	0.391
PN	T	18.8	2.05	16.31	1.47	−2.49	−2.082z	**0.037**
GF	T	20.92	2.46	20.24	2.84	−0.68	0.443	0.667
CP	T	11.34	0.61	11.72	1.07	0.38	−0.758	0.466
BA	T	11.48	0.74	9.78	0.86	−1.70	3.658	**0.004**
BP	T	11.59	1.24	13.34	0.93	1.74	−2.748	**0.021**
**Cellulose**								
All	–	25.20	10.98	24.29	19.91	–	0.798	0.442
Grass	G	36.57	5.41	35.95	6.05	–	0.220	0.837
Woody species	T	17.08	4.12	15.97	3.81	–	1.997	**0.047**
BN	G	29.59	2.28	28.17	1.03	−1.42	−1.121z	−0.262
AS	G	38.05	1.03	37.36	0.57	−0.69	1.431	0.183
DS	G	35.67	1.7	34.97	1.75	−0.7	−0.320z	0.749
EJ	G	35.05	1.13	34.36	0.89	−0.7	1.186	0.263
SU	G	44.48	1.21	44.91	4.36	0.43	−0.233	0.824
TS	T	21.7	1.57	23.34	1.66	1.64	−1.751	0.110
AA	T	9.57	1.41	9.11	1.67	−0.46	0.518	0.615
PN	T	22.18	1.25	18.15	1.02	−4.03	6.118	**<0.001**
GF	T	18.16	1.11	16.79	1.42	−1.37	1.864	0.092
CP	T	20.94	1.57	21.68	1.82	0.74	−0.754	0.468
BA	T	16.42	0.64	15.03	1.06	−1.40	2.761	**0.020**
BP	T	17.03	2.28	16.08	0.73	0.95	0.972	0.368
**Lignin**								
All	–	6.23	2.81	6.02	2.53	–	0.399	0.697
Grass	G	3.85	1.31	3.88	1.03	–	−0.031	0.976
Woody species	T	7.94	2.28	7.56	2.11	–	0.607	0.566
BN	G	2.06	0.47	2.21	0.27	0.16	−0.707	0.496
AS	G	5.05	0.56	5.07	0.34	0.02	−0.062	0.951
DS	G	2.89	0.41	3.94	0.41	1.06	−4.471	**0.001**
EJ	G	4.89	0.51	4.01	0.36	−0.87	−2.887z	**0.002**
SU	G	4.35	0.78	4.15	1.2	−0.2	0.346	0.736
TS	T	6.42	1.1	8.4	2.08	1.98	−2.065	0.066
AA	T	6.81	1.01	5.81	1.19	−1	1.565	0.149
PN	T	10.22	1.76	6.74	0.54	−3.49	4.647	**0.001**
GF	T	6.52	0.91	6.27	0.74	−0.24	0.51	0.621
CP	T	8.77	0.56	8.96	0.67	0.19	−0.531	0.607
BA	T	11.51	0.34	11.31	1.41	−0.2	0.336	0.749
BP	T	5.31	0.61	5.42	0.77	0.11	−0.284	0.783

**Notes:**

Mean diff, Differences in means between burned and control sites; PFG, Plant functional group (G, Grasses; T, Trees/woody species); SCC, Soluble cell compounds; grass species: AS, *Aristida stipitata*; BN, *Brachiaria nigropedata*; DS, *Digitaria seriata*; EJ, *Eragrostis jeffreysii*; SU, *Stipagrostis uniplumus*; tree species: AA, *Acacia ataxacantha*; BA, *Burkea africana*; BP, *Bauhinia petersiana*; CP, *Combretum psidiodes*; GF, *Grewia flava*; PN, *Philenoptera nelsii*; TS, *Terminalia sericea*.

All, All species; T, T-test; Z, Mann-Whitney-U-Test (indicated by “z”), Wilcoxon Test by*.

Significant differences (*P*) are indicated in bold.

**Table 4:** Concentrations (%) of secondary compounds in leaves of woody species found at two different sites.

	Control		Burned			T	*P*
	Mean	Std. Dev.	Mean	Std. dev.	Mean Diff.		
**Cond. Tannins**							
All	1.726	0.96	1.78	1.12	–	−0.339	0.746
TS	1.41	0.18	1.41	0.24	0	−0.014	0.989
AA	2.41	0.39	1.7	0.16	−0.72	4.167	**0.005**
PN	0	0	0	0	0	x	x
GF	1.23	0.18	1.47	0.17	0.24	−2.318	**0.043**
CP	1.82	0.27	1.51	0.44	−0.31	1.437	0.181
BA	2.99	0.33	3.46	0.45	0.47	−2.062	0.066
BP	2.15	0.9	2.9	0.48	0.75	−1.806	0.101
**Tot. Phenolics**							
All	4.43	2.46	4.61	1.85	–	−0.497	0.637
TS	7.34	0.73	6.55	0.77	−0.79	1.822	0.099
AA	5.03	0.57	5.02	0.65	−0.01	0.028	0.978
PN	0.88	0.06	1.63	0.25	0.75	−7.079	**0.001**
GF	2.33	0.27	2.84	0.51	0.52	−2.188	0.053
CP	7.21	0.5	5.9	0.76	−1.3	3.488	**0.006**
BA	5.29	1.19	6.25	0.98	0.96	−1.518	0.016
BP	2.97	1.15	4.06	0.33	1.09	−2.229	**0.050**

**Notes:**

Mean diff, Differences in means between burned and control sites; tree species: AA, *Acacia ataxacantha*; BA, *Burkea africana*; BP, *Bauhinia petersiana*; CP, *Combretum psidiodes*; GF, *Grewia flava*; PN, *Philenoptera nelsii*; TS, *Terminalia sericea*; All, All species; T, T-test.

Significant differences (*P*) are indicated in bold.

The comparisons for all compounds of all summarized species or each plant functional group showed no significant difference between the recently burned and control site, except from cellulose and ash concentrations of the leaves of woody species (Tables 2, 3).

### Effect of plant functional group and burning on plant chemistry

All models were significant and showed significant effects of the interaction between plant functional group × burning status and the species (Table 5), except for ash, hemicellulose and lignin. For these three compounds the effects of the interaction plant functional group × burning status were not significant.

**Table 5:** General Linear Models to test for the effect of burning and plant functional group (PFG) as well as plant species on different chemical compounds in leaves of woody species and grasses.

Chemical compound	Sum of square	*F*	*P*
**Nitrogen**			
Constant term	514.70	55.95	0.000
Burning * PFG	1.43	7.04	0.001
Species	91.99	90.37	0.000
**Ash**			
Constant term	2,025.42	71.82	0.000
Burning * PFG	2.26	2.219	0.113
Species	282.00	55.39	0.000
**SCC**			
Constant term	278,569.97	604.55	0.000
Burning * PFG	98.68	5.48	0.005
Species	4,607.87	51.13	0.000
**Hemicellulose**			
Constant term	80,365.14	459.30	0.000
Burning * PFG	11.38	2.03	0.135
Species	1,749.73	62.46	0.000
**Cellulose**			
Constant term	95,619.91	358.19	0.000
Burning * PFG	31.88	5.50	0.005
Species	2,669.55	92.17	0.000
**Lignin**			
Constant term	4,913.38	136.56	0.000
Burning * PFG	3.01	1.27	0.285
Species	359.79	30.24	0.000

**Note:**

Dependent variable: chemical compound, fixed factor: interaction between burning and plant functional group, random factor: (plant) species.

### Differences in plant response to fire between plant functional groups

**Figure 1:**
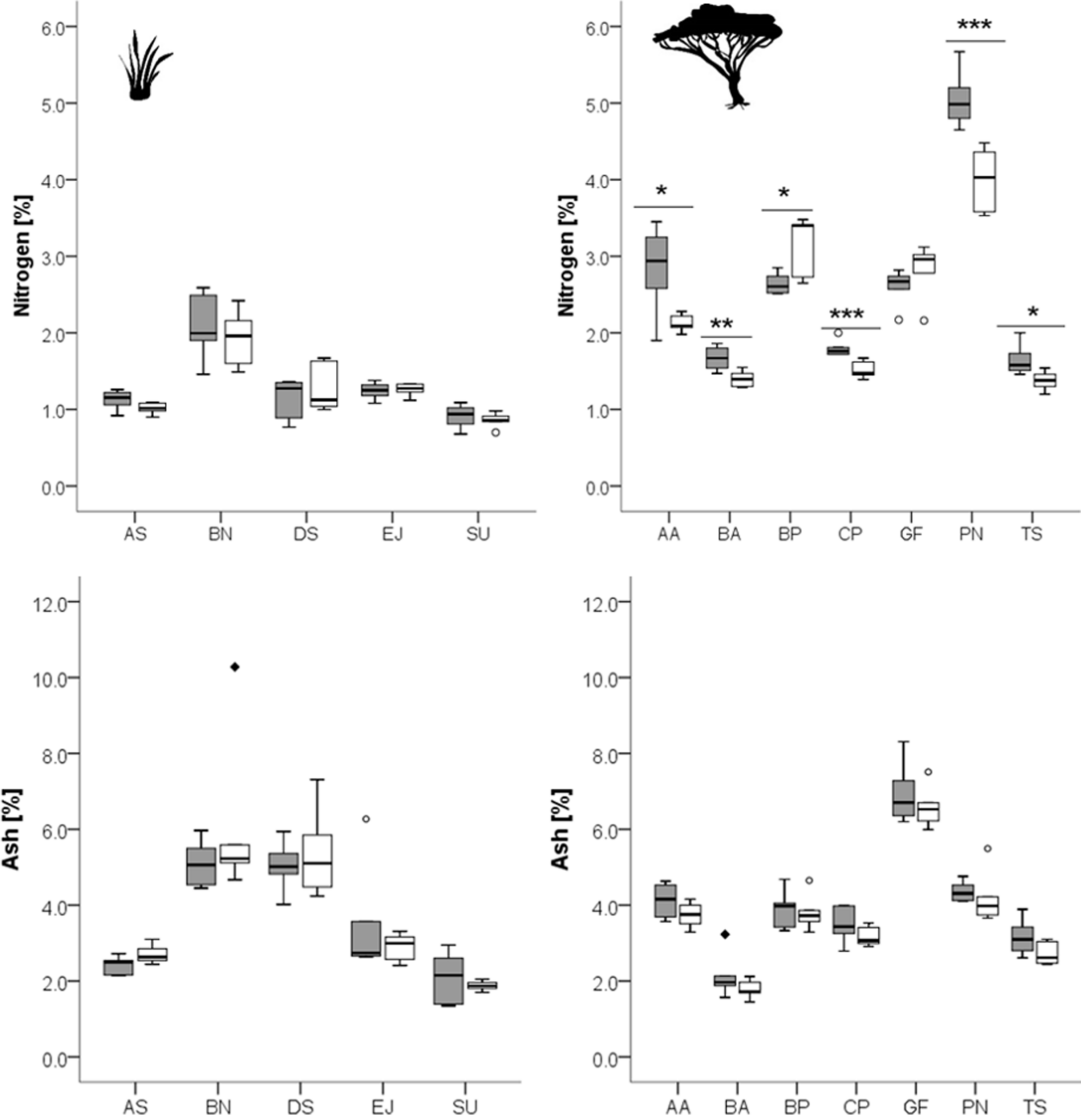
Concentrations of nitrogen and ash in grasses (left column) and leaves of woody species (right column) growing in the recently burned (grey) and control (white) site. Grass species: AS, *Aristida stipitata*; BN, *Brachiaria nigropedata*; DS, *Digitaria seriata*; EJ, *Eragrostis jeffreysii*; SU, *Stipagrostis uniplumus*; woody species: AA, *Acacia ataxacantha*; BA, *Burkea africana*; BP, *Bauhinia petersiana*; CP, *Combretum psidiodes*; GF, *Grewia flava*; PN, *Philenoptera nelsii*; TS, *Terminalia sericea*. Significant differences are indicated by *P ≤ 0.05; **P ≤ 0.01; ***P ≤ 0.001, exact *P*-values are given in Table 2. Boxes show medians, Q25 and Q75; whiskers are extremes and dots or squares indicate outliners.

**Figure 2:**
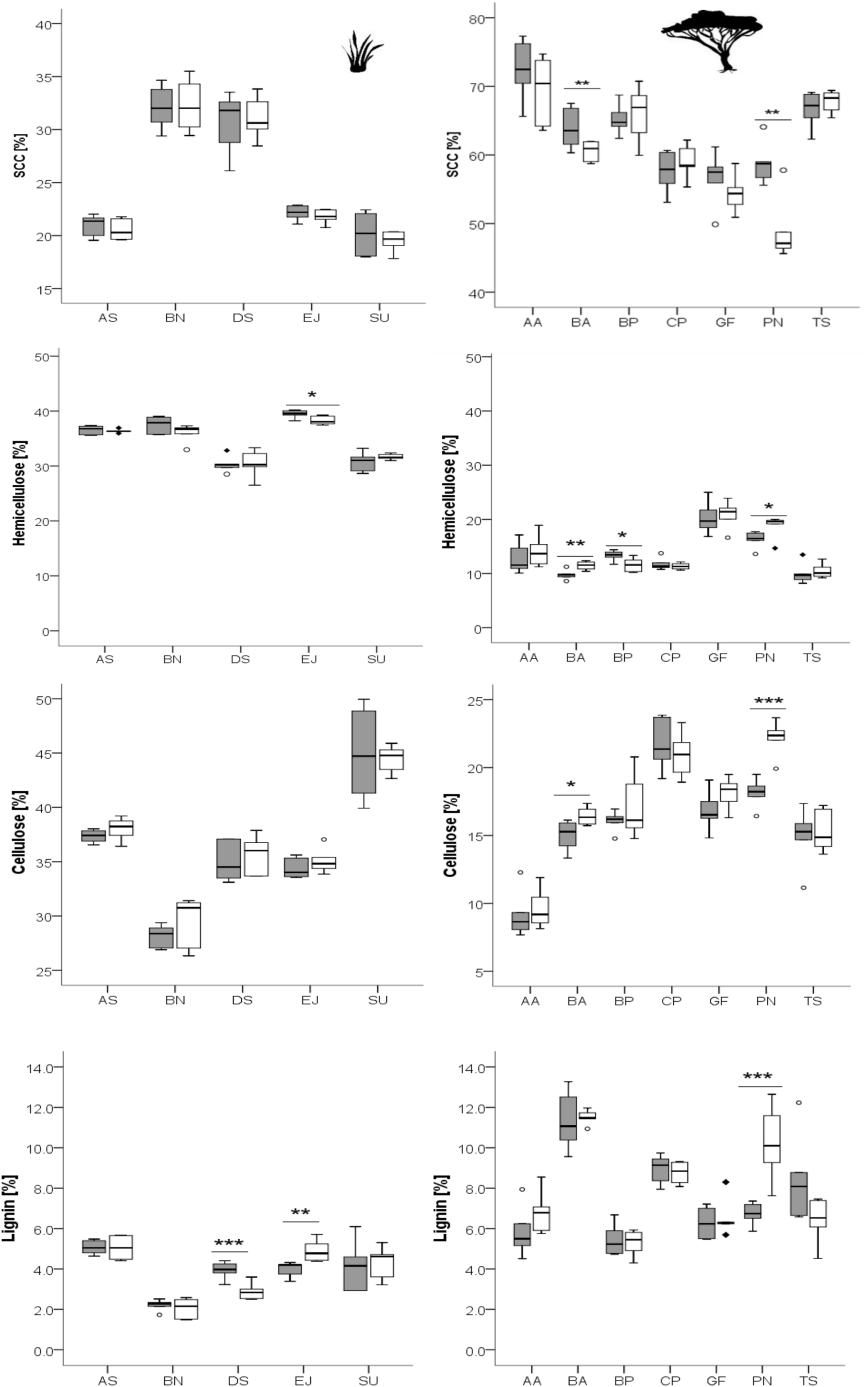
Concentrations of different fiber fractions in grasses (left column) and leaves of woody species (right column) growing in the recently burned (grey) and control (white) site. Grass species: AS, *Aristida stipitata*; BN, *Brachiaria nigropedata*; DS, *Digitaria seriata*; EJ, *Eragrostis jeffreysii*; SU, *Stipagrostis uniplumus*; woody species: AA, *Acacia ataxacantha*; BA, *Burkea africana*; BP, *Bauhinia petersiana*; CP, *Combretum psidiodes*; GF, *Grewia flava*; PN, *Philenoptera nelsii*; TS, *Terminalia sericea*. Significant differences are indicated by *P ≤ 0.05; **P ≤ 0.01; ***P ≤ 0.001, exact *P*-values are given in Table 3. Boxes show medians, Q25 and Q75; whiskers are extremes and dots or squares indicate outliners. SCC, Soluble cell compounds. Note the differences in units between graphs of cellulose and SCC for grasses and leaves of woody species.

**Figure 3:**
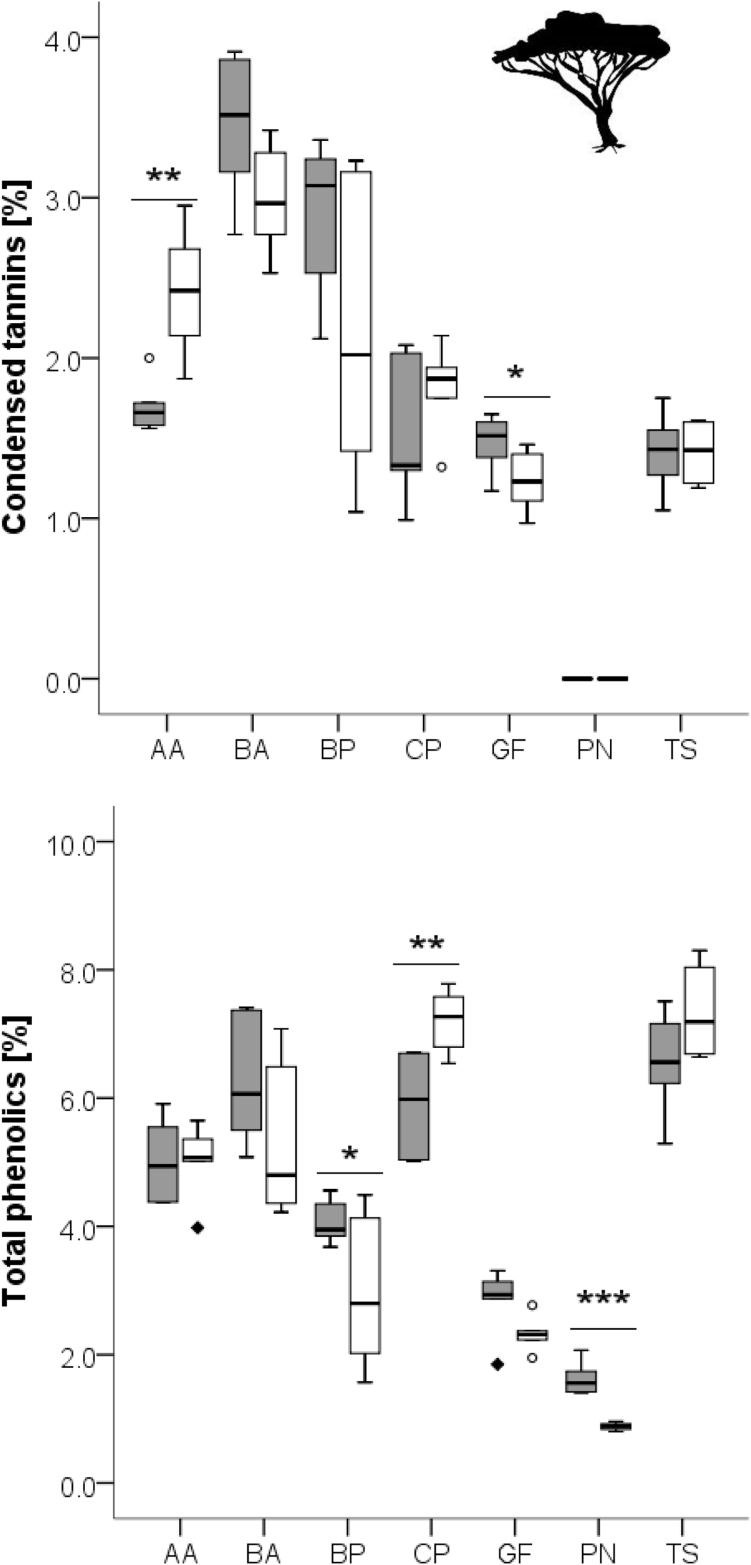
Concentration of total phenolics and condensed tannins in leaves of woody species growing in the recently burned (grey) and the control (white) site. Woody species: AA, *Acacia ataxacantha*; BA, *Burkea africana*; BP, *Bauhinia petersiana*; CP, *Combretum psidiodes*; GF, *Grewia flava*; PN, *Philenoptera nelsii*; TS, *Terminalia sericea*. Significant differences are indicated by *P ≤ 0.05; **P ≤ 0.01; ***P ≤ 0.001, exact *P*-values are given in Table 4. Boxes show medians, Q25 and Q75; whiskers are extremes and dots or squares indicate outliners.

For detailed results of the chemical analysis see Tables 2–4 and Figs. 1–3. Overall, the different grass species showed only weak differences in plant chemistry between recently burned and control site (Figs. 1, 2). Only *E. jeffreysii* showed significant differences in more than one of the measured compounds (hemicellulose and lignin, Table 3).

However, for woody species we found pronounced differences (Figs. 1, 2): for *P. nelsii* six of the measured eight compounds differed significantly between sites (for nitrogen see Table 2, SCC, for cellulose, lignin, hemicellulose see Table 3 and total phenolics see Table 5), and for *B. africana* four compounds differed significantly (nitrogen, and SCC, hemicellulose and cellulose). Furthermore, we found significant differences for *B. petersiana* (nitrogen, hemicellulose, total phenolics), *A. ataxacantha* (nitrogen, condensed tannins), and *C. psidioides* (nitrogen, total phenolics). Hence, we found different responses to fire for grasses and woody species.

We found an almost consistent pattern for woody species regarding nitrogen, with significantly higher concentrations of nitrogen in the leaves of woody species in the recently burned area compared to control site (*G. flava* and *B. petersiana* were the only exceptions, Fig. 1).

A similar tendency was found for SCC for four woody species, however the results were not always significant (Fig. 2, Table 3).

The trend of an opposite pattern was found for hemicellulose in most woody species with lower concentrations at the recently burned site. However, only two species were significant (*P. nelsii B. africana*, Fig. 2, Table 3), and *B. petersiana* was an exception with significantly higher concentration in the recently burned area (Fig. 2, Table 3).

Furthermore, we found a slightly higher concentration of ash of woody species in the recently burned area, but the results were not significant (Fig. 1, Table 2).

For grass species there was no such pattern found neither for nitrogen, SCC, nor for hemicellulose or ash (Figs. 1, 2).

The cellulose concentration seems to be slightly lower on the recently burned site for some of the woody species and most grass species, but only for two woody species the results were significant (*P. nelsii* and *B. africana*, Table 3).

For lignin we found rather inconsistent patterns for grass and woody species. There were significant differences between sites for the woody species *P. nelsii* and the grass *E. jeffreysii* (Table 3). Both had higher concentrations in the control area, in contrast to the grass species *D. sericea* which was significantly lower in the control area (Table 3).

Total phenolics and condensed tannins seemed to be differently affected by fire, and differences were species dependent (Fig. 3).

### Species specific plant response patterns

**Figure 4:**
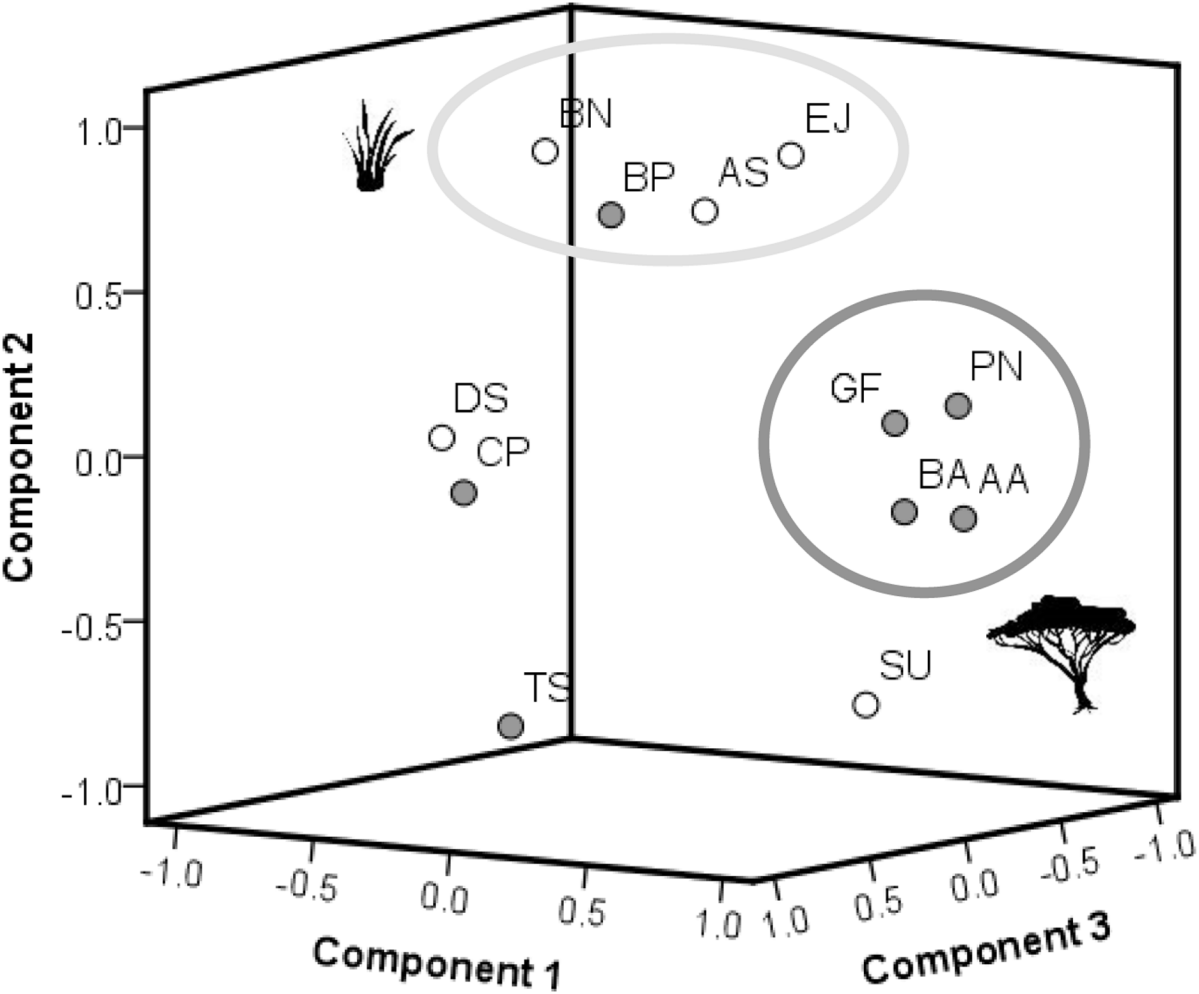
Results of the PCA (factor loadings). The PCA is based on differences in means of measured plant compounds (=mean concentration of all measured plant compounds of plants on recently burned site minus mean concentration of compounds of plants on control site, see Tables 2, 3). Woody species are indicated by dark grey dots, grass species by white dots. Grass species: AS, *Aristida stipitata*; BN, *Brachiaria nigropedata*; DS, *Digitaria seriata*; EJ, *Eragrostis jeffreysii*; SU, *Stipagrostis uniplumus*; woody species: AA, *Acacia ataxacantha*; BA, *Burkea africana*; BP, *Bauhinia petersiana*; CP, *Combretum psidiodes*; GF, *Grewia flava*; PN, *Philenoptera nelsii*; TS, *Terminalia sericea*: A. PCA of primary compounds of all plant species. Component 1–3 explained 96% of the total variance with component 1 (*x*-axis): 45%, component 2 (*y*-axis): 33% and component 3 (*z*-axis): 18% of the variance. The two distinct main groups are indicated by large circles. Members of each group show similar plant response, *e.g.*, dark grey circle represents mainly component 1. The light grey circle represents component 2.

The results of the PCA (Fig. 4) show the differences in chemical pattern (response to burning) between all plant species. The results indicate a separation between woody species and grasses in terms of how the chemical concentrations of all primary compounds combined differed between control and recently burned site. Four woody plant species (*A. ataxacantha, B. africana, G. flava, P. nelsii*) are found in one group. These woody species showed a similar chemical pattern. All four species show a positive difference in means for SCC and ash, as well as a negative difference for hemicellulose, cellulose and lignin. In addition, *A. ataxacantha, B. africana* and *P. nelsii* showed a positive difference for nitrogen. Interestingly, the grass species *S. uniplumus* plotted in close vicinity to woody species, as *S. uniplumus* shows a similar pattern to fire. The woody species *B. petersiana* was grouped together with grasses, as *B. petersiana* showed a positive difference in hemicellulose, cellulose and lignin, which is in contrast to the other woody species but more similar to grasses.

Located far from the main group of woody species in the PCA were *T. sericea* and *C. psidioides*, as they differ in SCC, cellulose, lignin compared to other woody species investigated. For grasses we found a more heterogeneous pattern. *A. stipidata* and *B. nigropedata* are found in one principle component as they only differed in their response in SCC. Both grass species showed a positive difference in means for nitrogen, hemicellulose and lignin (slightly higher in the recently burned area), and a negative difference for ash and cellulose (slightly lower in the recently burned area, Tables 2, 3). *E. jeffreysii* was also found in this principal component as it shows the same pattern for hemicellulose (positive) and cellulose (negative) but in contrast a negative difference in means for lignin, nitrogen and a positive difference for ash. The pattern for *D. seriata* is different to the other grass species and depends on differences of single compounds (species specific response).

## Discussion

In many ecosystems worldwide plants are known to respond to fire, and after a fire event plants are often preferred food for large herbivores, both domestic and wild (*e.g.*, Moe & Wegge, 1997; Fuhlendorf & Engle, 2004; Schindler, Fulbright & Forbes, 2004; Murphy & Bowman, 2007; Johnson et al., 2019). Thus, the attractiveness of plants in a post-fire area is likely attributed to enhanced food quality (Fryxell & Sinclair, 1988; Seagle & McNaughton, 1992; Parrini & Owen-Smith, 2010; Schindler, Fulbright & Forbes, 2004). The qualitative response of plants to a fire event is therefore interesting for the development of different management plans, *e.g.*, for farming or parks/nature reserves, as the occurrence of fire usually results in a subsequent increased utilization by (large) herbivores, which in-turn influences their distribution (Archibald et al., 2005; Archibald, 2008; Parrini & Owen-Smith, 2010; Uunona, 2014; Joubert et al., 2018; Petersson et al., 2020).

The results of our modelling approach showed an interactive effect of burning status and plant functional group on the plants’ concentrations of nitrogen, SCC and cellulose. In other words, the effect of fire on these plant compounds depended on which plant functional group the species belonged to. The results are against our expectations that after a fire changes in plant chemical composition should be similar for both functional groups. The non-significant interactions in our modelling approach involving the other three plant compounds (ash, hemicellulose and lignin) are likely explained by a more species-specific response in both plant functional groups.

The results of our modelling approach are underpinned by the results of our inter-specific comparisons. The comparisons reveal a rather homogenous response to fire for different woody species but almost no differences between the study sites for perennial grasses, with woody species maintaining a high nutritional value 2 years after burning whereas grasses did not, or only marginally differed in quality between the two sites.

Due to the high utilization of the recently burned site by large herbivores (Uunona, 2014) we assumed plants of both functional groups to have higher nitrogen concentration but also a higher mineral concentration (ash) in plants in the recently burned area (Ben-Shahar & Coe, 1992, but see review Felton et al., 2018). But contrary to our expectations, we found no significant differences between recently burned and control sites in mineral concentration (ash) in grasses, and woody species had only minimally higher concentrations in the recently burned area. More strikingly, we found that only leaves of woody species–not grasses–were higher in nitrogen in the recently burned area compared to the control area. Therefore, only our results for woody species are in accordance with one of our hypotheses (higher nitrogen concentrations in the recently burned area) and with the findings of Pellegrini et al. (2015), who found higher N values for woody species with a longer time lag to fire, but no such pattern for grasses. But our results are in contrast to the findings of Jensen, Michelsen & Gashaw, 2001, where grasses showed a distinct response in nutritional values shortly, 1 year after burning. Unfortunately, we did not measure plant chemistry immediately after burning. Therefore, we do not know, how plant chemistry changed in the time span between burning and sampling.

Conventionally, higher mineral and N availability in the soil lead to higher concentrations of these compounds in plants (Blair, 1997; Ball, Danell & Sunesson, 2000; Mbatha & Ward, 2010). However, Nghalipo et al. (2018), (see also Table 1) did not find higher concentrations in the upper soil on the same study sites. Therefore, the difference between the two functional groups concerning the nitrogen concentration in leaves cannot be solely explained by soil properties.

We rather suggest that our results can be due to physiological or morphological differences between grasses and woody plants, *e.g*., differences in the ability to utilize minerals (such as Ca, Mg, K, N) post fire. As the pattern of higher nitrogen in leaves of woody plants compared to grasses was not only true for N-fixing woody plants like those belonging to the Fabaceae family (*e.g., A. ataxacantha*) but also for other woody species, we suggest that the “response” pattern of relatively higher nitrogen concentration in the recently burned area found for most woody species may be due to the deeper root system of woody plants compared to the grass roots.

We further suggest only a short-term effect of fire on grasses resulting in higher values of plant nitrogen concentrations shortly after burning (*e.g*., Ojima et al., 1994; Jensen, Michelsen & Gashaw, 2001; Parrini & Owen-Smith, 2010) in combination with a relatively fast decline of nitrogen in the soil and the plants (Van de Vijver, Poot & Prins, 1999; Allred et al., 2011; McGranahan et al., 2014). This short-term effect can therefore not be detected 2 years after burning. Combustion at high temperature can lead to volatilization of nitrogen and to a conversion into inorganic N compounds in the soil, which will be lost by leaching during the first rain after burning. Both are major pathways of N loss in grassland ecosystems (Blair, 1997; Knicker, 2007). Therefore, in our case (2 years after burning) inorganic N may have been lost in the upper soil system and this process is pronounced because of the sandy soil in our study system (Nghalipo et al., 2018). Studies from burning show reduced N mineralization resulting in N limitations for plants (Blair, 1997; Turner et al., 1997; Fynn, Haynes & O’Connor, 2003; Strydom et al., 2019), especially as the organic matter of the surface is lost due to combustion (Ojima et al., 1994; Johnson & Matchett, 2001; Fynn, Haynes & O’Connor, 2003).

Contrary to grasses, the deep root system of trees/shrubs seems to not only be beneficial to utilize minerals (including inorganic N) that may have been washed down into the deeper soil layer, but maybe even more important to function as a storage for energy rich compounds (C-storage, Schutz, Bond & Cramer, 2009). This storage might enable woody plants resource allocation from root to shoot and a vigorous regrowth. The response of the investigated woody species is reminiscent of well documented plant response to browsing, where plants compensate by upregulating production of enzymes needed for photosynthesis and plant growth (Anderson, Fuhlendorf & Engle, 2006) and translocating nutrients from roots to shoots (Kruger & Reich, 1993), resulting in higher protein contents (McNaughton, 1984; McNaughton, Ruess & Seagle, 1988; Ruess & McNaughton, 1988; Archibald, 2008; Scogings, Hjältén & Skarpe, 2011), and larger leaves of woody plants with less structural compounds after a browsing event (Stolter et al., 2005; Stolter, 2008). Due to the regrowth process after burning we therefore suggest that lower concentrations of structural compounds (cellulose, hemicellulose and lignin), are caused by the higher productivity on a burned site combined with a more vigorous growth (Growth Differentiation Balance Hypothesis, Herms & Mattson, 1992), which is similar to the findings of Johnson et al. (2019) on western snowberry’s postburn regrowth. Cellulose was in lower concentrations in some woody species in the recently burned area, which may mirror a lower leaf toughness, especially for *P. nelsii* and *B. africana* (but also for *A. ataxacantha* and *G. flava*, but less pronounced). A similar but weaker pattern with lower concentrations in leaves of woody species on the recently burned site was found for lignin and hemicellulose. Meanwhile, the differences in grasses were more species specific. However, the loss of moribund old tussock parts due to burning (Amputu, Joubert & Mapaure, 2019) might still provide a better foraging circumstance compared to an unburned tussock patch, as the regrowing grass parts are more easily accessible than before the fire. The regrowth is highly attractive for herbivores feeding on perennial grasses in the burned area compared to the control site (Uunona, 2014), where moribund plant parts may cover new annual growth.

Furthermore, one can expect a lower concentration of tannins and phenolics as a response to fire as the available nitrogen and minerals should be rather utilized to growth of new photosynthetic tissue instead of a donation to defence mechanisms (Carbon Nutrient Balance Hypothesis and Growth Differentiation Balance Hypothesis, (Bryant, Chapin & Klein, 1983; Herms & Mattson, 1992; Scogings, Hjältén & Skarpe, 2011)), as protein and phenolic production compete for the common precursor phenylalanine (Donaldson, Kruger & Lindroth, 2006; Caretto et al., 2015). However, we only found two woody species with lower plant secondary metabolites concentrations in the leaves in the recently burned area (*A. ataxacantha, C. psidioides*). Nitrogen-fixation in woody species (*e.g., B. petersiana*, *B. africana, A. ataxacantha*) could be an opportunity to overcome the phenylalanine-bottleneck, as those species showed higher PSM concentrations in the recently burned site. But none of the trees with nitrogen fixation had higher nitrogen concentrations in the leaves and we also found non-nitrogen-fixing woody species (*G. flava*) with higher PSM. Therefore, we conclude a more species-specific response of PSMs to fire history. More species-specific studies are needed to elucidate the underlying mechanisms of plant chemical responses to fire, and to understand the physiological similarities of plant response to the two disturbances fire and herbivory.

## Conclusions

Two years after burning, the overall nutritional quality (*e.g.*, higher concentration of nitrogen, lower concentrations of structural carbohydrates) of leaves of woody species was higher on the recently burned site compared to the control site, but not for grasses. This indicates a longer lasting response to fire in woody species compared to grasses. Our results also indicate that the response of grasses to fire may be more species-specific and short-term than the responses of woody species. This emphasizes that, at least in our study system, the chemical response is more related to the plant species in focus than it is related to the plant functional trait “grasses”. We suggest that there is a need for more species-specific studies on plant responses to fire, conducted in various habitat types and under different fire regimes.

The higher nutritional quality of leaves of woody species in recently burned compared to the control area found in this study, provides an explanation to earlier observations that local browsers utilize burned sections disproportionately more (Uunona, 2014; Joubert et al., 2018). The fire may keep woody species in a prolonged rejuvenation phase (Amputu, Joubert & Mapaure, 2019) resulting in a positive feed-back loop for herbivores, especially browsers, similar to what is known from other study regions and large wild herbivores (Stolter, 2008). Therefore, woody species may still serve as an attraction in burned areas to various herbivores even when the fire has occurred 2 years earlier. This should be acknowledged in future management strategies.

## Supplemental Information

10.7717/peerj.12721/supp-1 Supplemental Information 1 Plant chemical composition data.

## Additional Information and Declarations

### Field Study Permissions

The following information was supplied relating to field study approvals (*i.e*., approving body and any reference numbers): The Ministry of Environment and Tourism of Namibia, Windhoek, allow the fieldwork to be conducted, the National Botanical Institute allow the transfer of the plant material.

### Data Availability

The following information was supplied regarding data availability: The raw measurements are available in the Supplemental File.
